# Neuro-Epigenetic Indications of Acute Stress Response in Humans: The Case of MicroRNA-29c

**DOI:** 10.1371/journal.pone.0146236

**Published:** 2016-01-05

**Authors:** Sharon Vaisvaser, Shira Modai, Luba Farberov, Tamar Lin, Haggai Sharon, Avital Gilam, Naama Volk, Roee Admon, Liat Edry, Eyal Fruchter, Ilan Wald, Yair Bar-Haim, Ricardo Tarrasch, Alon Chen, Noam Shomron, Talma Hendler

**Affiliations:** 1 Functional Brain Center, Wohl Institute for Advanced Imaging, Sourasky Medical Center, Tel Aviv, Israel; 2 Sackler Faculty of Medicine, Tel Aviv University, Tel Aviv, Israel; 3 Sagol School of Neuroscience; Tel-Aviv University, Tel Aviv, Israel; 4 Department of Neurobiology Weizmann Institute of Science, Rehovot, Israel; 5 Center for Depression, Anxiety and Stress Research, McLean Hospital, Harvard medical School, Boston, Massachussetts, United States of America; 6 Division of Mental Health, Medical Corps, IDF, Ramat Gan, Israel; 7 School of Psychological Sciences, Tel Aviv University, Tel Aviv, Israel; 8 School of Education, Tel Aviv University, Tel Aviv, Israel; Chiba University Center for Forensic Mental Health, JAPAN

## Abstract

Stress research has progressively become more integrative in nature, seeking to unfold crucial relations between the different phenotypic levels of stress manifestations. This study sought to unravel stress-induced variations in expression of human microRNAs sampled in peripheral blood mononuclear cells and further assess their relationship with neuronal and psychological indices.

We obtained blood samples from 49 healthy male participants before and three hours after performing a social stress task, while undergoing functional magnetic resonance imaging (fMRI). A seed-based functional connectivity (FC) analysis was conducted for the ventro-medial prefrontal cortex (vmPFC), a key area of stress regulation. Out of hundreds of microRNAs, a specific increase was identified in microRNA-29c (miR-29c) expression, corresponding with both the experience of sustained stress via self-reports, and alterations in vmPFC functional connectivity. Explicitly, miR-29c expression levels corresponded with both increased connectivity of the vmPFC with the anterior insula (aIns), and decreased connectivity of the vmPFC with the left dorso-lateral prefrontal cortex (dlPFC). Our findings further revealed that miR-29c mediates an indirect path linking enhanced vmPFC-aIns connectivity during stress with subsequent experiences of sustained stress. The correlative patterns of miR-29c expression and vmPFC FC, along with the mediating effects on subjective stress sustainment and the presumed localization of miR-29c in astrocytes, together point to an intriguing assumption; miR-29c may serve as a biomarker in the blood for stress-induced functional neural alterations reflecting regulatory processes. Such a multi-level model may hold the key for future personalized intervention in stress psychopathology.

## Introduction

When confronted with stress a cascade of psychophysiological responses are set in motion, while adaptive coping and post-stress recovery largely depends on optimal neuronal functioning, requiring precise and rapid gene expression changes. This may be achieved through post-transcriptional regulation via microRNAs (miRNAs) [1], with each miRNA targeting tens to hundreds of genes, overall regulating approximately half of the transcriptome [2, 3].

Several studies reported miRNA expression changes in the brain upon exposure to stress [4–7], also with relation to animal behavior [8, 9]. Psychological perturbations induce distinct gene expression changes also in humans [10]. Additionally, adverse experiences give rise to gene expression changes in circulating immune cells [11], corresponding with the well-recognized communication between the brain and immune system; peripheral blood mononuclear cells (PBMCs) share ~80% of the transcriptome with brain tissue [12, 13]. Therefore, miRNA expression signatures may extend beyond the brain, providing a useful peripheral biomarker of stress-responsiveness. Rapid expression changes in PBMCs were presented after brief exercise [14], yoga practice [15] and also after exposure to trauma, accompanying differential clinical outcomes, potentially harboring valuable prognostic information for identifying the onset of psychopathology [16–18]. Notably, a recent research demonstrated the feasibility and importance of unraveling the link between epigenetic regulation tested in blood and behaviorally and clinically relevant brain function in humans [19]. The intriguing relations between peripheral molecular changes, behavioral responses and brain function is beginning to be explored.

Brain-imaging studies implicate the medial prefrontal cortex as a core area for processing of emotional events [20, 21]. Its dorsal portions are involved in explicit appraisal, whereas ventral portions have a regulatory role. Through its interaction with other prefrontal and subcortical regions, the ventro-medial prefrontal cortex (vmPFC) plays a critical role in modulating and integrating physiological and behavioral aspects of stress-responses both in rodents [22, 23] and humans [24–27]. Indeed, stress-induced gene expression changes were evident particularly in the vmPFC [28–32]. However, debates continue as to how neurobehavioral changes occur with respect to genetic regulation.

In our study, we sampled PBMCs before and 3-hours after social-stress induction in the fMRI-scanner. Neural indications were obtained from a seed-based functional-connectivity (FC) analysis of the vmPFC. Behavioral indications were based on repeated subjective stress ratings. We hypothesized that variations in stress-responsive miRNAs, presenting variability in expression among participants, would correspond with processes of affect regulation manifested in the subjective reports and attributed to the vmPFC connections. Gaining such understanding could help reveal indicants of individual susceptibility to stress; providing a foundation for understanding the link between cellular responses and neural circuit functioning underlying neuropsychiatric symptoms.

## Materials and Methods

### Participants

The study was approved by the Tel Aviv Sourasky Medical Center (TASMC) ethical committee. 61 healthy male IDF soldiers (age 19–22) from the same unit, amidst the same military course, and before operational deployment participated in the study. All participants had normal or corrected-to-normal vision and provided written informed consent, approved by the ethical committee and conformed to the Code of Ethics of the World Medical Association (Helsinki Declaration). Molecular data was acquired from 49 participants, for which sufficient RNA samples were obtained. 4 participants were excluded from the fMRI data analysis due to signal artifacts (final sample N = 45). Participants had no reported history of psychiatric or neurological disorders, no current use of psychoactive drugs, no family history of major psychiatric disorders, and no previous exposure to abuse during childhood and/or potentially traumatic events before entering the study.

### Experimental Procedure

The protocol consisted of 4 phases: acclimation (15-min), 1st MRI scanning session (65-min), intermission (90-min) and a 2nd MRI session (60-min). During acclimation, participants were given a 15-min resting period, were introduced to the experimental procedure and their first blood sample for miRNA profiling was drawn. The second blood sample was obtained at the end of the experiment. MRI testing, in the 1st scanning session, included two 'rest' scans (5 min each) interspersed by a control task and an arithmetic social stress task. During 'rest' conditions participants were instructed to stare at a fixation point. The 2nd MRI session included a 'rest' scan and memory task.

### Stress elicitation task

Stress was induced using a serial subtraction arithmetic task [33–35]. Participants were instructed to continuously subtract 13 from 1022 for a period of 6 min, and respond verbally, while the experimenters monitored and communicated with each subject on-line, constantly demanding faster and more accurate performance. A timer appeared at the top left corner of the screen to indicate to the participant how much time had elapsed. The stress task was preceded by a non-stressful condition- backward counting from 1000 for a period of 6 min, without external monitoring.

### Behavioral and physiological data collection

**Fig 1:**
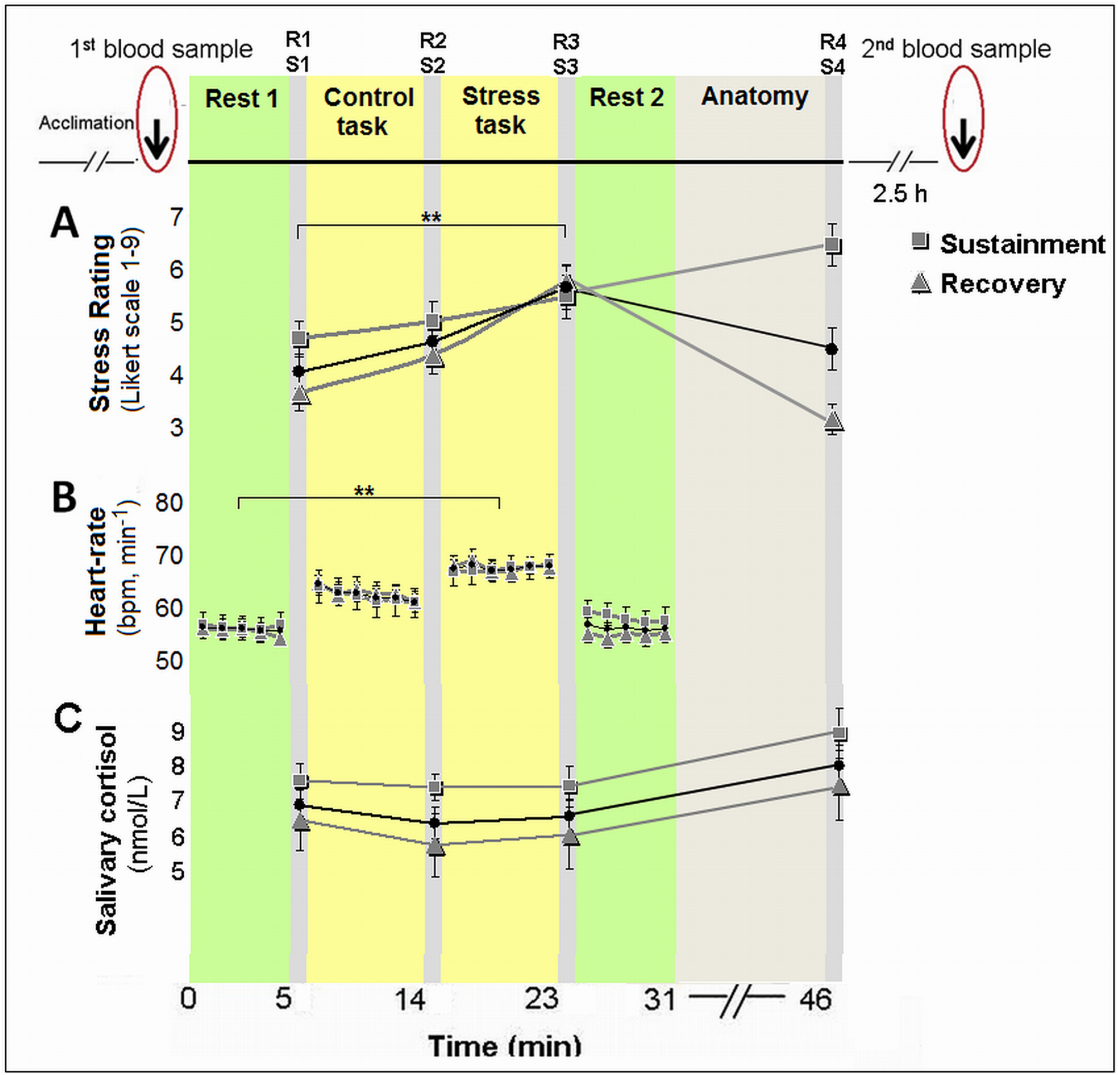
Experimental design, psychological and physiological responses to stress. Following the acclimation phase and the first blood sample drawn for miRNA expression, participants underwent a scanning session that included control (backward counting) and stress (serial subtraction) tasks; 3-hours following stress induction blood was drawn again. Elicitation of stress is shown by repeated subjective reports of stress levels (R1-4), heart-rate records and salivary cortisol samples (S1-4); for the whole cohort and for separate groups according to stress sustainment vs. recovery. The black line and circle represent the whole sample. ** p<0.001.

The psychological effect was evaluated through repeated self-reports of stress (on a 9 point Likert scale, marked as R); physiological effects were evaluated via continuous heart-rate measure and salivary cortisol (marked as S). Subjective stress and cortisol were sampled at 4 time points: after the first 'rest' scan [R1/S1], after the control task [R2/S2], after the stress task [R3/S3] and 20-min following a second 'rest' scan and an anatomical scan [R4/S4] (Fig 1, top panel).

To evaluate traces of stress observed in the self-reports, we calculated changes in the ratings 20-min after stress induction ended (R4), as compared to the ratings obtained immediately after the stress task (R3). We speculated that a decrease in the reported post-stress experience indicated more effective emotion regulation processing. Thus, participants were divided into two groups—*recovery* of subjective stress, presenting a reduction in stress ratings; and *sustainment* of subjective stress, presenting a lack of reduction (Δ> = 0).

### Endocrine Data Analysis

Salivary cortisol levels were assayed using Coat-A-Count radioimmunoassay (Siemens, Los Angeles, CA), inter- and intra-assay coefficient of variation (CV) 14.4%, 8.9%, respectively. Inter-assay % CVs of less than 15 and intra-assay % CVs of less than 10 are considered to indicate assays with good and reliable performance.

### Electrophysiological data collection and analysis

Electrocardiography (ECG) was recorded continuously via a BrainAmp ExG MRI-compatible system (BrainProducts, Munich, Germany). The sampling rate was 5000 Hz. Bipolar Ag/AgCl electrodes were attached to the sides of the chest. Preprocessing and RR-interval analysis included gradient artifacts removal using FASTR algorithm [36], implemented in FMRIB plug-in for EEGLAB [37]. R-peaks were detected using FMRIB toolbox, and corrected for misdetection (maximum correction rate across participants was 5.95%) and presence of ectopic beats. Finally, RR-intervals were used to derive a beats-per-minute index. Due to motion artifacts, 44 participants were included in the final heart-rate analysis, for which a reliable R-peak signal was detected.

### Blood sampling for miRNA profiling

Blood was drawn twice in EDTA-tubes: upon arrival at the MRI facility, following acclimation, and at the end of the second scanning session, approximately 3-hours after the stress task. The interval was based on the notion that gene expression takes a few hours to complete, along with previous evidence regarding rapid expression changes in PBMCs in response to real-life events [15, 17, 18]. The blood samples were delivered within 1-hour to Shomron's lab in temperature-controlled chambers.

### Study procedure for miRNA extraction and profiling

**Fig 2:**
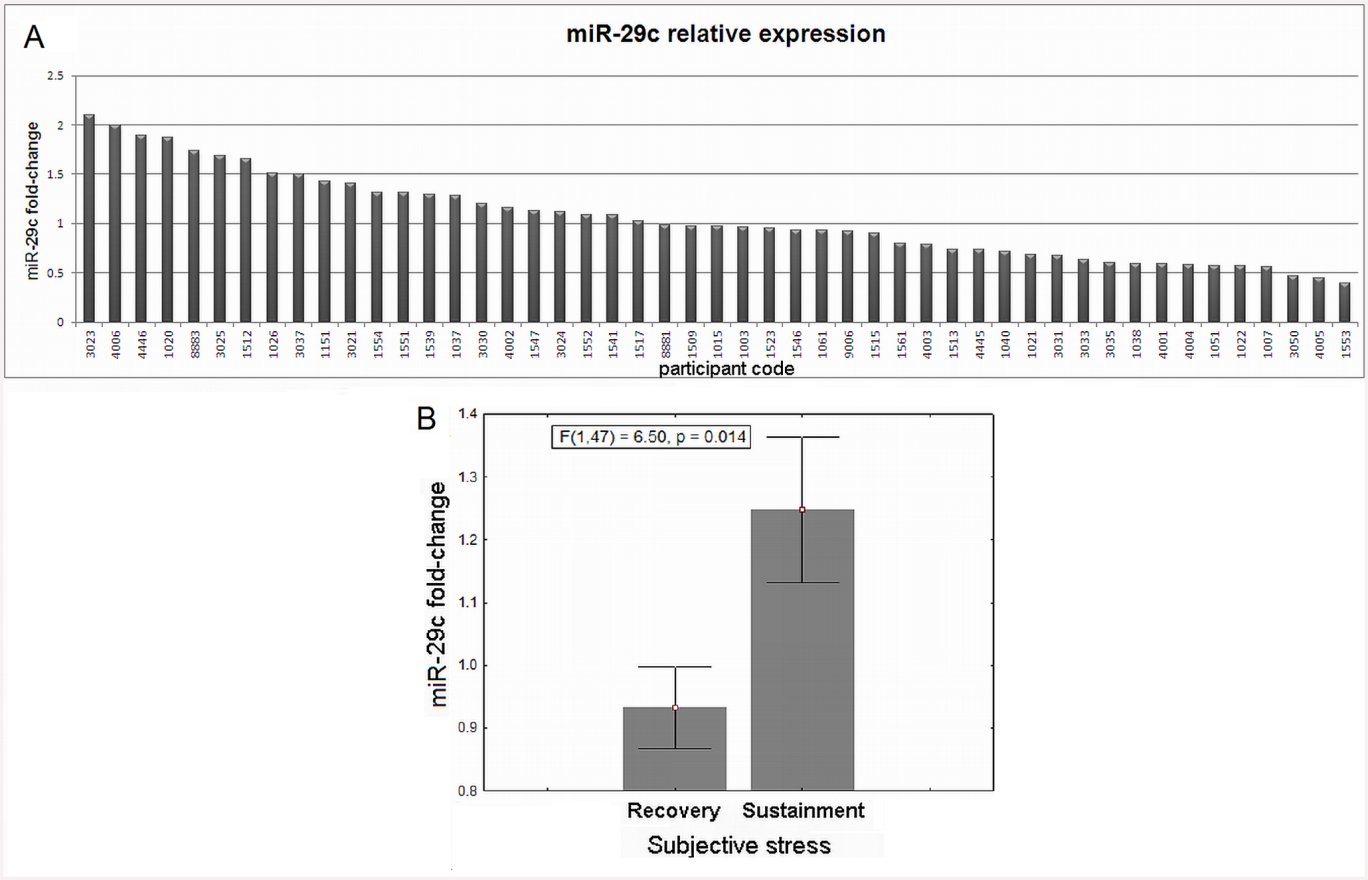
Stress-induced change in miR-29c and sustained subjective stress. **A**. MiR-29c stress-induced fold-change (axis y) for all participants (coded in axis x); **B**. ANOVA analysis between groups revealed that increase in miR-29c expression was related to sustained stress.

MiRNAs were extracted from blood samples using Trizol (see below). We first focused on 10% of the sample (6 individuals), randomly picked for initial miRNA profiling. This RNA was ran on a TaqMan Low Density array (TLDA, see below). Relevant miRNAs presenting *stress-induced change* were selected according to two parameters: all Cts were required to be between 15 and 35, and a fold-change variance > 2 was needed between individuals. The analysis revealed 6 miRNAs that presented *stress-induced change*: hsa-let-7b, miR-342-3p, hsa-miR-20b, hsa-miR-345, hsa-miR-30c, hsa-miR-29c. Out of these, we further examined hsa-miR-29c, based on the previously found expression in the human prefrontal cortex and its suggested involvement in CNS disorders [38, 39]. Consequentially, we ran a series of TaqMan real-time PCR assays to deduce miR-29c expression in all participants (Fig 2A).

### Specific methods for microRNA analysis

#### 1. Lymphocytes extraction

Lymphocytes extraction was carried out not later than 1-hour after blood collection; Every 10 ml whole blood sample was divided into 2 tubes containing 25ml EL buffer (Qiagen), gently mixed and incubated on ice for 20 min. Tubes were centrifuges for 8-min in 2000rpm at 4°C. Supernatant was removed and 10 milliliters of EL buffer was added. Tubes were mixed, incubated on ice for 15-min and centrifuges for 8-min in 2000rpm at 4°C. Supernatant was removed and 1 milliliter of EL buffer was added. Tubes were mixed and centrifuges for 5-min in 5500rpm at 4°C. Supernatant was removed and 200 microliters of EL buffer was added. Tubes were mixed and centrifuges for 5-min in 2000 rpm at 4°C. Supernatant was removed and pellets were kept in -80°C for later use.

#### 2. RNA extraction

RNA was extracted from the lymphocytes pellet using Trizol (Life Technologies), according to manufacturer's instructions, and kept in -80 degree Celsius. RNA quality assessed with a gel electrophoresis.

#### 3. miRNAs profiling

1 μg of total RNA was used to generate cDNA using the TaqMan Low-Density Arrays (TLDAs), which are quantitative real-time-polymerase chain reaction (RT—PCR) assays, based on specific stem—loop primers, each is complement to a mature miRNA. These primers are found in a mixture which generates a multiplex PCR reaction (megaplex). Profiling and analyzing procedures included the following work: first-strand cDNA was made with High Capacity cDNA kit (Life technologies). Complementary DNA (cDNA), RNase-free water and TaqMan Universal PCR Master Mix (No AmpErase UNG; Life technologies) was then infused into a loading port on Human TLDA card A, centrifuged twice and sealed according to the manufacturer’s instructions. PCR amplification was done on an ABI Prism 7900HT Sequence Detection System under the following conditions: 2 min at 50 degree Celsius, 10 min at 95 degree Celsius, 40 cycles of (30 s at 95 degree Celsius and 1 min at 60 degree Celsius). Results were analyzed with SDS software (Applied Biosystems / Life technologies). miRNA relative levels were calculated based on the comparative threshold cycle (Ct) method (see RQ calculation below). We used miR-425-5p for normalization, which according to our results did not change under stress (see S1 Table). Reactions were run on an Applied Biosystems 7900HT Fast Real-Time PCR System.

#### 4. Quantitative Real-Time PCR

10 nanograms of RNA were used to generate cDNA using the TaqMan MicroRNA Reverse Transcription Kit and TaqMan MicroRNA qPCR assay (Life Technologies using triplicate reactions. We used miR-425-5p for normalization. Reactions ran on StepOnePlus Real-Time PCR System (ABI).

#### 5. Relative Quantification (RQ) Calculation

Normalization was achieved by reducing the Ct of each miRNA from the average Cts of all miR-425-5p replicates ΔCt = (CtmiRNA—CtmiR-425-5p). For each miRNA, we reduced the normalized Ct in the first sample (T_0_ –before stress) from the normalized Ct in the second sample (T_1_ –after stress) to create ΔΔCt values (T_1_-T_0_). RQ number is calculated by 2 exponent the remainder from the last step (2^−ΔΔCt^). *P*-value was calculated for the relative levels using the two-sample unequal variance *t*-test and values were adjusted for multiple testing by the false discovery rate (FDR) method of <0.1 [40].

### MiR-29 expression in mice subjected to a social defeat stress

To further investigate the link between mir-29 and stressful experience, the expression of miR-29 family (miR-29a, b and c) was further acquired and analyzed from brain tissue of socially defeated mice (see S1 File).

### fMRI data acquisition and analysis

Brain scanning was performed on a 3T (GE, HDXt) MRI scanner with an 8-channel head coil. Functional imaging was acquired with gradient echo-planar imaging (EPI) sequence of T2*-weighted images (TR/TE/flip angle: 3000/35/90; FOV: 20 × 20 cm; matrix size: 96 × 96) in 39 axial slices (thickness: 3 mm; gap: 0 mm) covering the whole cerebrum. fMRI data analysis was performed with SPM5 (Wellcome Department of Imaging Neuroscience, London, UK). Preprocessing of the fMRI data included correction for head movements (subjects with movement above 2 mm were discarded) via realignment of all images to the mean image of the scan using rigid body transformation with six degrees of freedom, normalization of the images to Montreal Neurological Institute (MNI) space by co-registration to the EPI MNI template via affine transformation, and spatial smoothing of the data with a 6 mm FWHM. Finally, the first 6 images of each functional resting scan were rejected to allow for T2* equilibration effects.

The vmPFC ROI was determined as follows: We first conducted an activation analysis. For each participant, the blood—oxygen level-dependent (BOLD) responses were modeled in a general linear model (GLM) prepared with the two conditions of control and stress tasks. A second level random effects group analysis (RFX) reveled increased vmPFC activity in the [stress-control] contrast at p<0.05, small volume corrected cluster utilizing an anatomical mask of the mPFC that included both sides of the medial frontal, orbital and rectal gyri and the anterior cingulate cortex, defined from the WFU Pickatlas toolbox for SPM [41]. Next, FC analysis was conducted for the functionally defined vmPFC cluster as a seed ROI. A mean time series across voxels in the seed ROI was calculated for each participant using the MarsBaR software. GLM analyses were performed between the ROI time series and the time series for each brain voxel. To reduce the effect of physiological artifacts and nuisance variables, 6 motion parameters, cerebrospinal fluid, and white matter signals were introduced as covariates in the design matrix. Next, a second level random effects group analysis (RFX) was conducted. Significant clusters that altered FC with the vmPFC seed ROI in [stress-control] contrast were identified at a threshold of p = 0.0005, FDR corrected for multiple comparisons, and anatomically validated with the WFU Pick Atlas Tool [41]. Beta weights were extracted and averaged across all voxels within each functional area. Two participants were excluded from this analysis as outliers due to beta values exceeding ±2.5 Std from group average.

### Mediation analysis

Mediation was tested in a standard three variables path model, using SPSS INDIRECT macro script [42], in which the indirect effect was considered significant if its 95% bootstrap confidence intervals from 1000 iterations did not include zero at p = 0.05.

## Results

### Stress elicitation

The paradigm yielded substantial stress reactivity at the whole group level, by measures of subjective stress, heart-rate and cortisol response [*black line and circle*, see also [34]]. A significant main effect of time was found for subjective stress-ratings (F(3, 180) = 17.562, p<0.001, two-sided); while post-hoc analyses revealed an increase in ratings in response to stress (R3), as compared to the two previous measures (R1 and R2, Tukey's honest significant difference (HSD), p’s<0.001), and a decline to baseline following the second rest period (R4, p<0.001, Fig 1A). The means and standard-deviations (in parentheses) of R1, R2, R3 and R4 were 3.69 (1.82), 4.21 (1.96), 5.34 (1.9) and 4.08 (2.39), respectively. Notably, the four measures of subjective stress were within the normality range (values of Skewness and Kurtosis were within the range of ±2 standard errors). Heart-rate (beats-per-minute) analysis also revealed a significant main effect of time (F(3, 129) = 38.88, p<0.001, two-sided), while post-hoc analyses revealed an increase in heart-rate in response to stress, as compared to pre-stress conditions (Tukey's HSD, p<0.001), and a decrease to initial levels during the second rest period (p<0.001, Fig 1B). The means and standard-deviations (in parentheses) of the 4 heart-rate measures (in *bpm*) were 57.97 (9.36), 65.25 (10.44), 69.39 (9.60), 58.99 (9.82), respectively. For salivary cortisol, we found a marginally significant main effect of time (F(3, 171) = 2.46, p = 0.064, two-sided). Post-hoc analysis revealed a peak in cortisol levels in the final sample (S4), as compared to post 'rest 1' sample (S1, Tukey's post-hoc analysis; p = 0.057, see Fig 1C). To note, a significant correlation was found between the subjective stress rating immediately post-stress (R3) and the (log transformed) cortisol AUCi (area under the curve increase, r = 0.28, p = 0.035).

### The subjective experience—post-stress recovery vs. sustainment groups

24 participants (39.3%) presented subjective stress *sustainment* with no reduction in self-reported stress ratings 20-min after the stress task had ended (Fig 1).

No significant difference was found in subjective stress ratings between the groups in samples R1-3 (p's = 0.40, 0.54, 0.81, respectively; Fig 1). Additionally, a repeated measures analysis revealed no between-group differences in both cortisol (p = 0.203) and heart-rate (p = 1.06) measures (Fig 1B and 1C). The subjective stress *sustainment* group had slightly, yet insignificantly higher cortisol levels at all time-points, S1-4. Our results of a correlation between subjective stress immediately after the stress task (R3) and cortisol AUCi, on the one hand, yet no such relation for the sustained stress measure, on the other hand, is in line with previous research indicating a relationship between psychological and physiological stress measures only during stress manipulation (Trier Social Stress Test) and not pre- and post- exposure [43].

### MiR-29c individual expression

Changes in expression levels of miR-29c for all participants is presented in Fig 2A. The overall mean (standard-deviation) fold-change was 0.98 (1.08). 27 participants presented a decrease in miR-29c post-stress expression, while 22 participants presented an increase. Mean (standard-deviation) fold-change was 0.73 (0.18) for decreases and 1.44 (0.32) for increases in expression.

### Correspondence between mir-29c expression change and subjective stress

The division into stress *sustainment* (n = 18 subjects with molecular data, 36.7%) vs. *recovery* (n = 31, 63.3%) was related to the miR-29c fold-change (Fig 2B). An ANOVA analysis revealed a larger miR-29c expression change in the stress *sustainment* group (F(1, 47) = 6.5, p = 0.014, two-sided), with a large effect size (Cohen's d = 0.72). A marginally significant correlation was found between the miR-29c fold-change and individual changes in stress ratings in R4-R3 (r = 0.27, p = 0.064). No relationship was found between miR-29c expression and cortisol responsiveness [repeated-measures ANOVA: p = 0.67; and Pearson correlation with AUCi: p = 0.86].

Our further investigation in mice subjected to a social defeat stress revealed a 1.8 fold increase in expression of miR-29 family in the PFC of stressed mice compared to controls (see S1 File).

### Neural correlates of stress induction

**Fig 3:**
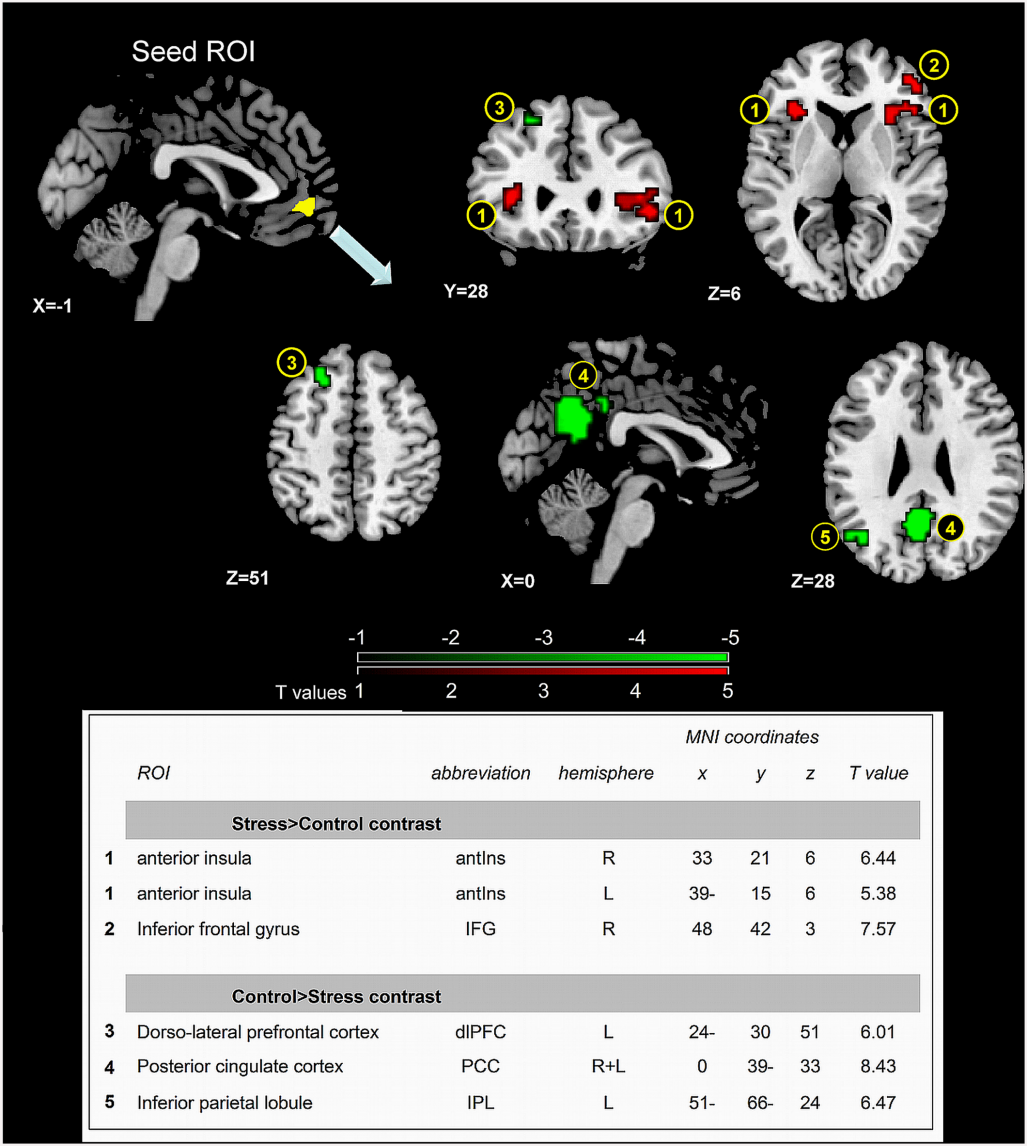
Stress-induced alterations in vmPFC FC. Areas that altered FC with the vmPFC seed ROI when contrasting stress vs. control, p(FDR corrected) = 0.0005. (1) Bilateral aIns (2) right IFG (3) dlPFC (4) PCC (5) left IPL. T-score scale is shown at the bottom, with red representing increased FC and green decreased FC. The table presents peak voxels and corresponding T values.

Fig 3 presents the areas which altered FC with the vmPFC cluster during stress induction. The analysis revealed enhanced FC with the bilateral anterior insula (aIns, 1) and right inferior frontal gyrus (IFG, 2) and decrease in FC with the dorso-lateral PFC (dlPFC, at Brodmann area 8, 3), posterior cingulate cortex (PCC 4) and inferior parietal lobule (IPL, 5) (see table in Fig 3 for peak coordinates and t values).

### miR-29c shows dynamic relations with vmPFC functional connections

**Fig 4:**
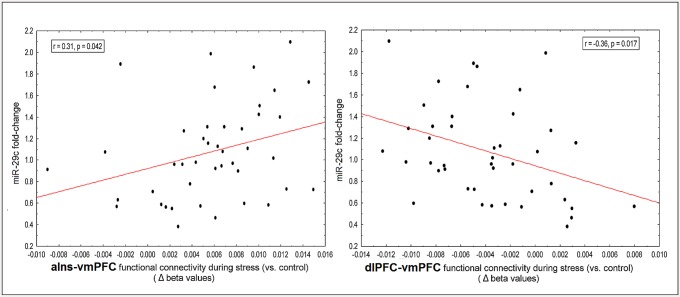
Correlations between miR-29c fold-change and vmPFC functional connections. **A**. miR-29c fold-change was positively correlated to vmPFC FC with the aIns; **B**. miR-29c fold-change was negatively correlated to vmPFC FC with the dlPFC.

A positive correlation (r = 0.31, p = 0.042, two-sided, Fig 4A) was found between individual miR-29c expression changes and the degree of change in vmPFC-aIns FC (during stress compared to control), and a negative correlation between individual miR-29c expression changes and vmPFC-dlPFC FC (r = -0.36, p = 0.017, two-sided, Fig 4B).

Notably, no correlation was found with regard to vmPFC coupling with the other clusters; right IFG, left IPL and PCC (p = 0.50, 0.70, 0.52, respectively).

### Relationship between vmPFC FC, mir-29c expression and subjective stress

**Fig 5:**
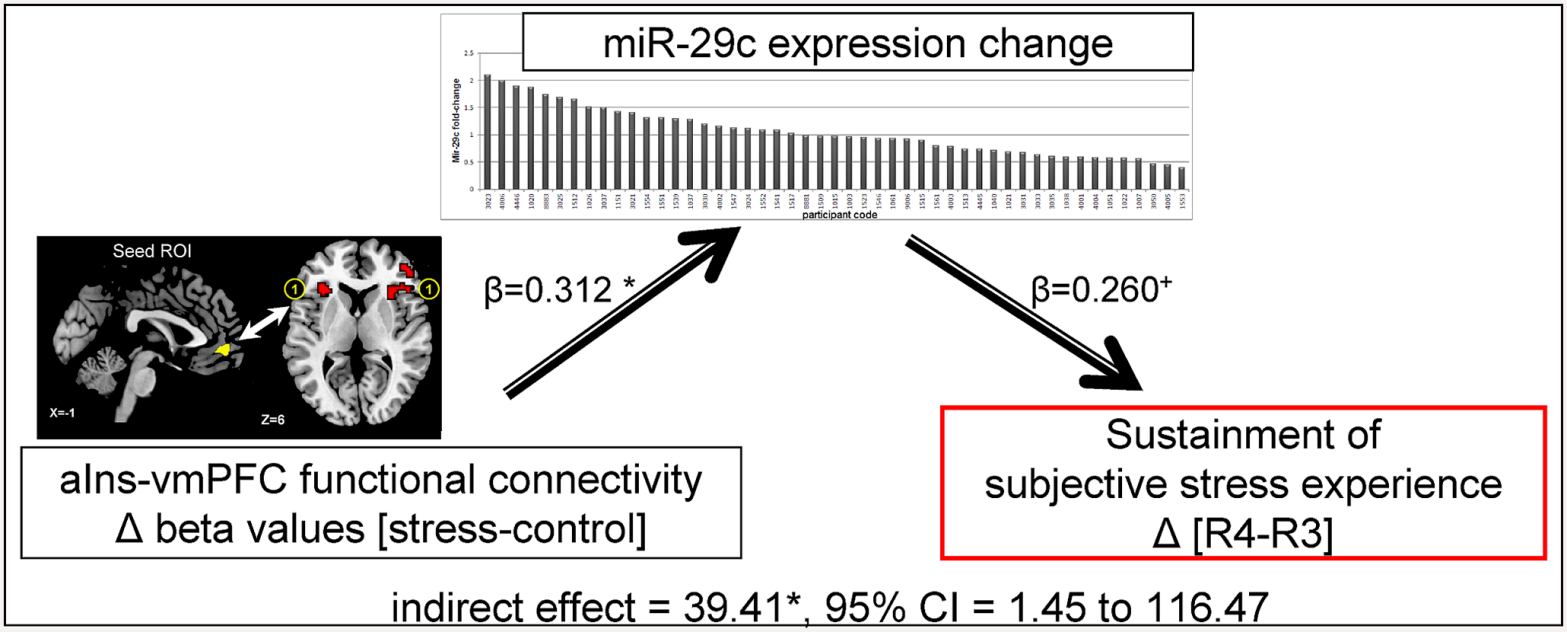
Mediating role of miR-29c in stress sustainment. The illustrated mediation model depicts a significant indirect path from vmPFC-aIns FC during the stress task (compared to control, delta beta values) to the change in subjective stress (in R4, 20-min after stress, compared to R3, immediately after stress), through miR-29c fold-change. Specifically, enhanced vmPFC-aIns FC led to higher reported stress levels through increases in miR-29c expression. Beta values are shown next to arrows indicating each link in the analysis. *p<0.05, ^+^p = 0.064.

Our results indicate that increased miR-29c expression is linked with both enhanced vmPFC-aIns FC during stress (Fig 4A) and subjective stress sustainment (Fig 2). Due to the temporal trajectory of these neural and behavioral features, we further explored the possibility that vmPFC-aIns FC during stress has an indirect effect on post-stress subjective reports (R4-R3) by the engagement of miR-29c expression change. To test this, we performed a mediation analysis [42], indicating that enhanced vmPFC-aIns FC during stress indeed led to a lasting subjective stress experience via enhanced miR-29c expression (indirect effect = 39.41*, 95% CI = 1.45 to 116.47, Fig 5). We likewise examined vmPFC-dlPFC FC and found no significant mediating effect (indirect effect: -44.81, 95% CI = -139.57 to 3.19). Notably, we also tested whether it is the vmPFC-aIns FC that mediates an indirect path from the level of expressed miR-29c to the subjective stress experience and found no significant mediating effect (indirect effect: -0.21, 95% CI = -0.86 to 0.13).

## Discussion

Our study indicates the feasibility of detecting specific miRNA changes in PBMCs shortly following stress induction in healthy humans, further assessing their relationship with neuronal and psychological indices. Together our findings point to a potential neuro-molecular marker for the subjective experience of stress.

### A possible molecular marker of individual stress response

Of the 6 miRNAs that presented *stress-induced changes* varying among participants, we focused on miR-29c, previously shown to be expressed in the human prefrontal cortex and involved in psychopathologies such as schizophrenia and bipolar disorder [39], and brain disorders such as Alzheimer's [44], Huntington's [45] and Parkinson's [46] diseases. MiR-29 family members have been acknowledged as epigenetic components that mediate neuropathological processes in response to environmental stress factors [47], and as such have been suggested as potential noninvasive biomarkers for diagnosis and prognosis of neuropsychiatric disorders [38]. We present rapid responsiveness of miR-29c to stress induction in PBMCs of healthy humans. Although the change in miR-29c expression is relatively modest, it could exert a meaningful impact on cell physiology (for example: [48, 49], see S1 Table).

We further present the novel relationship between the epigenetic level of response and the subjective experience of stress; enhanced miR-29c expression corresponded with sustained stress feelings (Fig 2B). The final ratings were obtained following 20-min of 'resting' time, during which participants were not engaged in any tasks. Nonetheless, ~40% of our sample showed persistent high stress ratings, as compared to their immediate post-stress report. This is particularly intriguing since they were not required to regulate their affective state and didn't know they would need to reassess their stress state.

The importance of microRNA families lay in their common sequence or structure, which promotes similar functionality [50]. Measuring the expression of miR-29 family (miR-29a, b, c) in the PFC of mice (see S1 Fig) demonstrated a stress-related increased expression of miR-29b, and further implies the cross species contribution of miR-29 as a family to stress response.

### Neural effectors of the molecular marker of stress responsiveness

Following the finding of correlation between the miR29c and the enduring of perceived stress we speculated that stress sustainment may have resulted from a failure in spontaneous regulation and thereby the molecular-behavioral association was driven by changes in relevant neural circuits. Indeed, we found that fold changes in miR-29c were greater on the one hand with increased vmPFC-aIns connectivity and on the other with decreased vmPFC-dlPFC connectivity during stress induction. This opposite pattern demonstrates a relationship between neural and epigenetic changes in humans which might correspond to stress regulation mechanism (Fig 4). The aIns in particular has been indicated as a core region involved in salience detection, integration and filtering of relevant emotional information [51], engendering awareness of feelings [52], and somatic representation [53], with evidence of its hyper-responsiveness in anxiety-prone individuals [54]. The aIns involvement in salience processing has been repeatedly suggested to be modulated via reciprocal connections with anterior ventral region in the PFC [52, 55–57]. Most important, the mPFC in particular was shown to be co-activated with the aIns during various manipulated cognitive states [58] and during responses to aversive relative to neutral stimuli [59]. Of particular interest, with regard to the possible role of this functional connectivity for adaptive regulating of stress, is the finding of enhanced mPFC-aIns FC following repeated exposure to traumatic memory [60] and during self-appraisal relative to semantic tasks [61], supporting a speculation that increased mPFC-aIns FC connectivity is involved in stressful self-appraisal process. This idea nicely corresponds with our mediation analysis results indicating that vmPFC-aIns FC enhancement is linked to sustainment of subjectively estimated stress (i.e. 20 min following the stress induction) via the neuro-molecular fold change of miR-29c (Fig 5). Together our findings support the role of both vPFC connectivity and epigenetic changes in self-related regulatory mechanism of stress feelings.

The additional finding of decreased vmPFC-dlPFC FC related to increased miR-29c expression may reflect the neuro-epigenetic regulation of both the sustained emotional response and the parallel disturbance of cognitive processing. A similar reduction in vmPFC-dlPFC FC was previously shown in the context of enhanced cognitive load; argued to be related to increased need to allocate attentional/cognitive processes, possibly to eliminate potential emotional processing interference [62]. Of particular interest is the proposed role of the dlPFC in conscious/explicit emotional evaluation [63, 64], and its link to cognitive appraisal of anticipatory anxiety [65]. Studies point to the up-regulation of miR-29c in schizophrenia and bipolar disorder, both involved in affective and executive control deficits [39]. Moreover, post-mortem studies on patients with schizophrenia report altered miR-29c expression in the dlPFC [66, 67]. Yet, in the current context of acute-stress the relation between enhanced mir29c and dlPFC function is linked to reduced FC with the vmPFC; possibly suggesting a relationship between miR-29c expression and the failure in its recruitment for emotion regulation via a more cognitive process.

Our results provide a neuromolecular account for inter-individual differences in post-stress recovery. It is yet unclear how miR-29c mediates this brain-behavior relationship, though one possible mechanism is through the miR-29 family's abundant expression in astorcytes [68]. Astrocytes are considered key players in synaptic plasticity regulation in different brain areas [69]. Dynamic astrocytic-neuronal interactions have important functional consequences due to modifications in extracellular ionic homeostasis, neurotransmission, gliotransmission, and ultimately neuronal function at the cellular and system level [70]. Further animal studies should explore the exact path of such neuromolecular interaction in stress regulation.

### Limitations

With regard to the temporal effect of miR-29c, we assume that stress-induced expression changes may occur minutes after induction, thus impacting one’s subjective experience. Previous evidence indeed points to rapid turnover as a common property of neuronal miRNAs [71]. Secondly, we assume that the relationship between miR-29c expression and neural connectivity is due to parallel expression patterns between peripheral blood and relevant brain regions. However, this should be determined empirically. We encourage further research extending from surrogate PBMC to brain regions of interest. We rely solely on miRNAs identified to—date; the platform we use interrogates 384 predefined miRNAs (from 'miRbase' database). An alternative approach could be to use 'Deep Sequencing' technology that allows the identification of both known and novel miRNAs that may be expressed in our setting. From the neuroimaging perspective, our interpretation of regulation processes might be based on reverse inference, thus we encourage further research investigating whether different stress regulation techniques might have an effect on miR-29c expression along with the perceived subjective stress.

### In summary

Our combined results point to miR-29c as a mediator of psychological stress, prompted by enhancement of vmPFC-aIns FC, simultaneously linked to decreased vmPFC-dlPFC FC and the assumed diminishment of evaluative-cognitive capacity needed to cease the emotional response. These findings, along with the presumed localization of miR-29c in astrocytes, lead to an intriguing notion: miR-29c may serve as a biomarker in the blood for plastic stress-induced alterations in functionally-distinct neural circuits, reflecting affective-cognitive regulation processes. Identifying components of epigenetic control related to regulatory brain function may provide the foundation for a new era in stress research and the future development of individually tailored early treatments for anxiety-related pathologies.

## Supporting Information

S1 Fig miR-29a, b, c levels in the PFC of mice subjected to social defeat paradigm. (TIF)

S1 File miR-29a, b, c in mice subjected to a social defeat paradigm. (DOCX)

S1 Table Raw TLDA data for miR-29c and its endogenous control miR-425. (DOCX)

## Abbreviations

PBMCs: peripheral blood mononuclear cells
FC: functional connectivity
fMRI: functional magnetic resonance imaging
miRNA/miR: microRNA
aIns: anterior insula
dlPFC: dorso-lateral prefrontal cortex
vmPFC: ventro-medial prefrontal cortex
