# Supplementary figures and images for: Neuro-Epigenetic Indications of Acute Stress Response in Humans: The Case of MicroRNA-29c
Source: PLoS One. 2016 Jan 5;11(1):e0146236. doi: 10.1371/journal.pone.0146236 (PMC4711717; doi:10.1371/journal.pone.0146236)

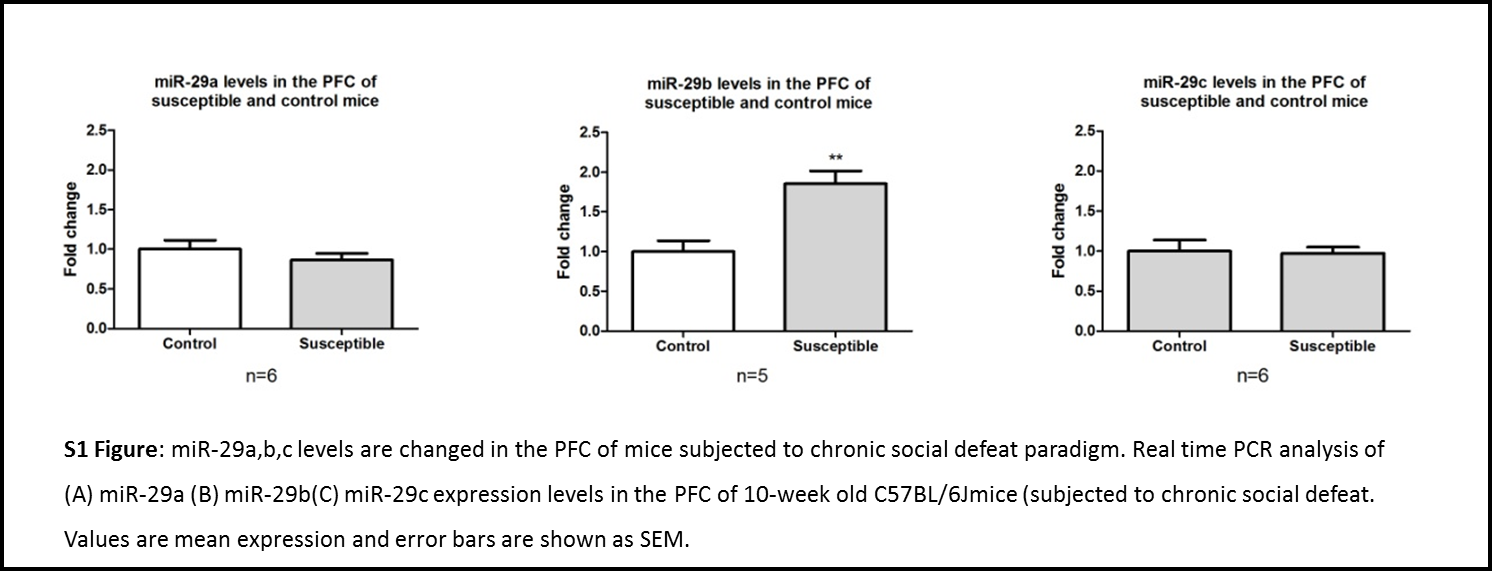

Supplement: S1 Fig — (TIF) [file pone.0146236.s001.tif]
