# Supplementary material for: Neuro-Epigenetic Indications of Acute Stress Response in Humans: The Case of MicroRNA-29c
Source: PLoS One. 2016 Jan 5;11(1):e0146236. doi: 10.1371/journal.pone.0146236 (PMC4711717; doi:10.1371/journal.pone.0146236)
Supplement: S1 File — (DOCX) [file pone.0146236.s002.docx]

**Supporting Information**

**S1 File: miR-29a,b,c in mice subjected to social defeat**

***Chronic social defeat in mice***

### 10-week old C57BL/6J mice were subjected to a social defeat protocol as previously described (1). Briefly, the mice were placed in a home cage of an aggressive ICR mouse and allowed to physically interact for five minutes. During this time, the ICR mouse attacked the intruder mouse and the intruder displayed subordinate posturing. A perforated clear Plexiglas® divider was then placed between the animals and the mice remained in the same cage for 24 hours to allow sensory contact. The procedure was then repeated with an unfamiliar ICR mouse for each of the 10 consecutive days. Control mice were housed in the same room as the social defeat mice but were escorted out of the room during the five minute interaction with the ICR. Control mice were handled daily and housed 2 in a cage with a perforated clear Plexiglas® divider placed between the 2 mice. The PFC punches were taken as part of a different experiment in which the social defeat paradigm was also used. The PFC that were not used for that experiment were selected to be used in this one, thus there was no statistical method in which the number of animal was selected.

***Microdissection of brain tissue***

Brain samples were collected from social defeat and control mice 8 days after the end of the social defeat protocol. Tissue collection and processing was as previously(2). Briefly, after removing the brain and placing it on an acryl 1 mm brain matrix (Stoelting, cat# 51380), slices of 2 mm were taken using standard razor blades (GEM, 62-0165) based on designated anatomical markers. Blunted syringes at different diameters were used to extract the different brain nuclei from slices removed from the matrix.

***miRNA RT-qPCR expression analysis***

Quantitative miRNA expression was acquired and analyzed using a step one thermocycler (Applied Biosystems), using primers that were designed for specific miRNA. RNA samples were assessed using miScript Reverse transcription kit and SYBR^®^Green PCR kit (Qiagen) according to the manufacturer's guidelines. U6 snRNA was used as internal control. Student's two tailed t-test was used for the analysis of the results. Mice were excluded from the analysis if they had values higher than AVG+2*SD or lower than AVG-2*SD.

Results

***MiR-29 expression in mice:*** Expression levels of miR-29 family in mice PFC are presented in Fig S1. MiR-29a and miR-29c average expression levels did not change in 6 social defeat treated mice compared to 6 control mice, but miR-29b was about  1.8 fold elevated in 5 social defeat treated mice compared to 5 control mice (t(8)=3.843 p=0.005). Values are normally distributed and the variance is similar between compared groups.

### References:

1. Krishnan V, Han MH, Graham DL, Berton O, Renthal W, Russo SJ, et al. Molecular adaptations underlying susceptibility and resistance to social defeat in brain reward regions. Cell. 2007;131(2):391-404.

2. Sztainberg Y, Kuperman Y, Tsoory M, Lebow M, Chen A. The anxiolytic effect of environmental enrichment is mediated via amygdalar CRF receptor type 1. Mol Psychiatry. 2010;15(9):905-17.
