# Supplementary material for: Neuro-Epigenetic Indications of Acute Stress Response in Humans: The Case of MicroRNA-29c
Source: PLoS One. 2016 Jan 5;11(1):e0146236. doi: 10.1371/journal.pone.0146236 (PMC4711717; doi:10.1371/journal.pone.0146236)
Supplement: S1 Table — (DOCX) [file pone.0146236.s003.docx]

**Supporting Information**

**S2 Table: The raw data of the TLDA for miR-29c and its endogenous control miR-425**

|  |  |  | **Endogenous control** | **marker** |
| --- | --- | --- | --- | --- |
|  |  | **Detector** | **hsa-miR-425-4380926** | **hsa-miR-29c-4395171** |
| **Ct** | **1037** | T0 | 28.29 | 30.12 |
|  |  | T1 | 27.87 | 28.86 |
|  | **1515** | T0 | 27.56 | 28.14 |
|  |  | T1 | 27.19 | 28.06 |
|  | **1517** | T0 | 26.50 | 27.55 |
|  |  | T1 | 27.49 | 24.18 |
|  | **1026** | T0 | 26.96 | 24.09 |
|  |  | T1 | 27.53 | 28.09 |
|  | **1512** | T0 | 26.73 | 26.37 |
|  |  | T1 | 26.85 | 24.64 |
|  | **1551** | T0 | 26.55 | 21.31 |
|  |  | T1 | 26.88 | 29.74 |
| **RQ** | **1037** | T1-T0 |  | 0.814 |
|  | **1515** | T1-T0 |  | 0.093 |
|  | **1517** | T1-T0 |  | 0.004 |
|  | **1026** | T1-T0 |  | 1.784 |
|  | **1512** | T1-T0 |  | 20.492 |
|  | **1551** | T1-T0 |  | 3.620 |
